# Multiparametric Monitoring in Equatorian Tomato Greenhouses (I): Wireless Sensor Network Benchmarking

**DOI:** 10.3390/s18082555

**Published:** 2018-08-04

**Authors:** Mayra Erazo-Rodas, Mary Sandoval-Moreno, Sergio Muñoz-Romero, Mónica Huerta, David Rivas-Lalaleo, César Naranjo, José Luis Rojo-Álvarez

**Affiliations:** 1 Departamento de Eléctrica y Electrónica, Universidad de las Fuerzas Armadas, Av. General Rumiñahui s/n, Sangolquí 171-5-231B, Ecuador; drrivas@espe.edu.ec (D.R.-L.); canaranjo@espe.edu.ec (C.N.); 2 Departamento de Teoría de la Señal y Comunicaciones, Sistemas Telemáticos y Computación, Universidad Rey Juan Carlos, 28943 Fuenlabrada, Spain; sergio.munoz@urjc.es (S.M.-R.); joseluis.rojo@urjc.es (J.L.R.-Á.); 3 Departamento de Ciencias Exactas, Universidad de las Fuerzas Armadas ESPE, Av. General Rumiñahui s/n, Sangolquí 171-5-231B, Ecuador; mjsandoval@espe.edu.ec; 4 Center for Computational Simulation, Universidad Politécnica de Madrid, Boadilla del Monte 28660 Madrid, Spain; 5 Carrera de Telecomunicaciones, Universidad Politécnica Salesiana, Cuenca 010105, Ecuador; mhuerta@ups.edu.ec

**Keywords:** wireless sensor networks, tomato, greenhouse, ZigBee, WiFi, DigiMesh, Libelium^TM^

## Abstract

In recent years, attention has been paid to wireless sensor networks (WSNs) applied to precision agriculture. However, few studies have compared the technologies of different communication standards in terms of topology and energy efficiency. This paper presents the design and implementation of the hardware and software of three WSNs with different technologies and topologies of wireless communication for tomato greenhouses in the Andean region of Ecuador, as well as the comparative study of the performance of each of them. Two companion papers describe the study of the dynamics of the energy consumption and of the monitored variables. Three WSNs were deployed, two of them with the IEEE 802.15.4 standard with star and mesh topologies (ZigBee and DigiMesh, respectively), and a third with the IEEE 802.11 standard with access point topology (WiFi). The measured variables were selected after investigation of the climatic conditions required for efficient tomato growth. The measurements for each variable could be displayed in real time using either a laboratory virtual instrument engineering workbench (LabVIEW${}^{\mathrm{TM}}$) interface or an Android mobile application. The comparative study of the three networks made evident that the configuration of the DigiMesh network is the most complex for adding new nodes, due to its mesh topology. However, DigiMesh maintains the bit rate and prevents data loss by the location of the nodes as a function of crop height. It has been also shown that the WiFi network has better stability with larger precision in its measurements.

## 1. Introduction

The agricultural zone in Ecuador is one of the leading sectors of the country economy. According to the Survey of Surface and Agricultural Continuous Production (ESPAC 2015) [1], the total national agricultural labor was 5.67 million hectares, of which 0.95 million were dedicated to the cultivation of varieties that are harvested at specific times of the year, known as transient products. The species *Solanum lycopersicun*, popularly known as tomato, is a plant native to the Andean region of South America, and it is one of the high production transient products in the provinces of Azuay, Carchi, Chimborazo, Loja, and Tungurahua. In 2015, the survey reported an annual production of 47,837 metric tons. This vegetable is one of the most cultivated worldwide due to its high nutritional content, which makes it a food consumed daily [2]. The most outstanding tomato varieties are Graciela, Daniela, Dominique, Bright, Katherine, and Electra, and all of them are grown in greenhouses [3]. It can be grown in open fields in tropical areas and valleys, and also in Andean regions under greenhouse conditions.

The greenhouses for growing tomatoes can be located from sea level up to 3200 m, and the crop requires an air temperature of 18–30 ${}^{\circ }$C, which can be controlled with proper management of greenhouse climate control systems. The climatic conditions of the crop significantly influence its efficient growth, and, in the case of the tomato, the most relevant variables are air temperature, air humidity, wind direction, solar radiation, soil moisture, and CO${}_{2}$. Their inadequate management can cause diseases and pests, and it can reduce the product size [4]. Greenhouses are used to minimize the influence of adverse factors limiting production and crop quality. They can support environmental variables and make efficient use of water [5], hence achieving better optimization in agricultural production. Although not all geographic areas meet the conditions required to grow tomato, there is a need for technological help to provide viable conditions for it. Research and development in science have introduced management systems for agricultural greenhouse which keep the indoor climate controlled for suitable crops growth [6,7]. The crops grown under greenhouses can be affected by adverse environmental conditions, such as high solar radiation, high air temperature, low air relative humidity during the day, high air relative humidity at dawn and dusk, poor ventilation, and limited CO${}_{2}$ concentration, which generally occur by aspects related to the timing day, the geographical area climate, or the greenhouse material type. Therefore, it is essential to include high-precision systems for the environmental monitoring both inside and outside of the greenhouses, in order to avoid the occurrence of the above-mentioned adverse climatic conditions. The implementation of this systems will minimize the producer’s economic losses due to the plants deterioration or death [8]. In recent years, the agricultural sector has benefited from the use of monitoring networks, which are used to increase the production efficiency [9,10]. Data corresponding to environmental conditions, such as air temperature, wind speed, wind direction, soil moisture, and chemical and physical soil properties (e.g., pH), are usually acquired [11].

Today, wireless technologies are a relevant complement to the implementation of monitoring systems, because they have advantages such as low power consumption, adaptable network topology, economic maintenance, capacity of expansion with new nodes, or ability to operate in harsh environments [12,13,14,15]. The usual disadvantages in these networks are security and electromagnetic interference, which in the case of monitoring systems of greenhouses can be considered as secondary aspects. This is due to the fact that the type of information that is transmitted does not require a high level of encryption, and the network can be configured in a channel of low saturation. The main wireless technologies currently used for monitoring greenhouses are Radio Frequency combined with GSM [16,17,18,19], WiFi [20,21,22], and ZigBee [23,24,25,26,27,28]. At present, the latter two technologies are widely used in WSNs, both have strengths and weaknesses, and their applicability depends or the type of information to be transmitted [29,30]. Few works have been developed in the field of WSNs for monitoring tomato greenhouses, and most of them focus on the analysis of air temperature and air humidity by using networks with star [31] and mesh [32] topology. In these studies, the objective is the design of the network nodes, as well as the design of human–machine interface (HMI) for the visualization of each variable. However, those studies do not analyze all the climatic variables that influence the tomato growth. There are very few comparative studies of the performance of ZigBee and WiFi technologies applied in WSNs for agriculture, and they do not always analyze in detail which of them is the most efficient in terms of configuration complexity, of transmission speed, or of energy consumption.

The objective of this work is to design and implement the hardware and software of the nodes of three WSNs, with different communication technologies, for the wireless monitoring of environmental variables in tomato greenhousesr to analyze and compare the benefits and limitations of each network, and to identify which of them yields the best performance for the optimal monitoring of agricultural environments. For this purpose, the following methodology was addressed. Three WSNs were designed and implemented: (1) ZigBee technology in star topology; (2) ZigBee with mesh topology (referred to here as DigiMesh); and (3) WiFi technology (access point topology). The environmental variables air temperature, air relative humidity, CO${}_{2}$ concentration, luminosity, wind direction, wind speed, solar radiation, and ultraviolet radiation (UV) were monitored in real time. The networks were in test mode for three months, and time data transmission rates were studied in different scenarios during this time. Processing, storage, and visualization of data were engineered in LabVIEW${}^{\mathrm{TM}}$ software, and a mobile application was implemented for smartphones with Android operating system. Our case study was a tomato greenhouse located in the town of Rumipamba of Navas, Canton of Salcedo, and Province of Cotopaxi, Ecuador. In the companion papers to this work, we also analyzed the dynamics of the energy consumption signals in each node and network, as well as the time-varying signals of the monitored environment variables [33,34].

The rest of this first article is organized as follows. Section 2 summarizes the state of the art, focused on communication technologies applied to greenhouse monitoring. Section 3 presents the software and hardware designed for the deployment of the three WSNs, as well as the HMI development and the mobile application, as tools for the visualization of monitored variables. Section 4 describes the results of the analysis of the prescriptions of each network. Finally, Section 5 presents the discussion and conclusions of this first part.

## 2. Related Work

Monitoring networks in greenhouses can be implemented through wired or wireless technologies. Wired networks communicate through wires, which connect them to computers and to other devices that form networks, whereas wireless networks consists of the interconnection of devices through radio-wave propagation technologies, without the need for structured wiring. Both technologies have strengths and weaknesses, and their use depends on the type of application. From the scientific literature review, not many studies can be found about the implementation of monitoring networks for greenhouses, and sometimes they use wired communication technologies such as CAN bus [35,36], MODBUS [37], and RS-485 bus [38]. The positive aspects of wired networks are larger bandwidth, maximum possible performance, and higher transmission speed, whereas the negative aspects are complex wiring structure, fixed network topology, high cost of installation and maintaining, high power consumption, and limitations to increase nodes. Wired technology is rarely used for greenhouse monitoring because of the indicated limitations. In the field of the monitoring of agricultural environments, wireless communication networks are increasingly used because their implementation does not require the installation of wiring over the crop. The most applied communication technologies for this purpose are ZigBee and WiFi, since their performance is high, in terms of reach, scalability and energy efficiency, and in addition they can be readily coupled to the user needs.

As the cost trend for sensors and wireless communication infrastructure goes down, more producers are implementing these systems. Monitoring of environmental variables through WSN allows the farmer to know the real-time weather status, its use is practical and effective, and they are an easily scalable option when adding new communication nodes [39]. The geographical location and type of crop determine the appropriate climatic conditions for greenhouses, hence contributing to rapid growth and without the presence of pests that cause disease. For example, farmers in the Andean region of Ecuador face the problem of frost, which occurs on nights when the air temperature near the surface of the ground decreases to 0 ${}^{\circ }$C or lower, for a time greater than four hours, causing partial or total destruction of the crops [40]. Recent research in the field of precision agriculture has shown that the use of information and communication technologies optimizes the crop monitoring and its automation.

During the last years, WSNs have represented one of the most suitable and widely used options for this purpose. The remarkable evolution of WSNs has allowed the implementation of new systems for sensing, processing, and data communication from remote locations, in real-time variables such as air temperature, air relative humidity, luminosity, or wind speed. A significant amount of scientific literature has focused on greenhouse monitoring systems, and Table 1 summarizes some studies in this area, showing that ZigBee is the technology used in most research cases, while WiFi and LORA are sparsely applied. Most jobs use ZigBee wireless technology in the 2.4 GHz band, and focus on the hardware and software development of network nodes, but there are few networks configured in mesh topology. In addition, all the reported works consider air temperature and air relative humidity as environmental conditions for the productivity of crops in greenhouses. The literature review concludes that there is little basic research analyzing the behavior of WSN networks in different topologies, and that WiFi and LORA technologies are scarcely applied.

## 3. Proposed System for Tomato Greenhouse Monitoring with WSNs

**Figure 1:**
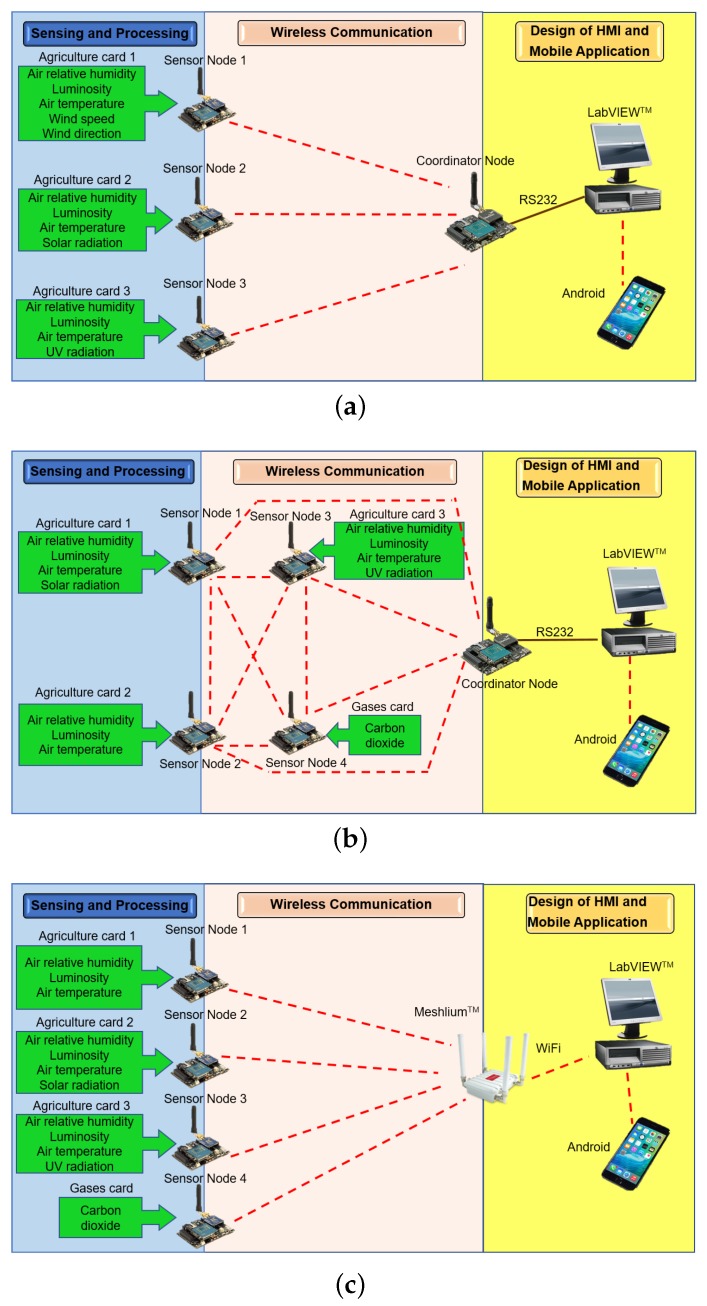
Schemes of the sensors and network topology of the studied WSNs: (**a**) ZigBee; (**b**) DigiMesh; and (**c**) WiFi.

To identify the performance of the ZigBee and WiFi standards for monitoring greenhouses, three WSNs were implemented. The number of network nodes, the type of installed sensors, and the configured topology are shown in the block diagrams of Figure 1. In this section, we provide the system design and implementation to monitor environmental variables inside the greenhouse. We describe next the methods and elements used for the analyzed WSNs. First, the suitable ranges of the variables that influence tomato growth are described, as well as the sensing, packaging, and processing techniques that were used. Second, the relevant hardware features and configuration parameters of the ZigBee, DigiMesh, and WiFi networks are described. Third, the design of the HMI and web application for variables visualization are explained. Finally, the physical characteristics of the greenhouses, the location of the elements of the networks, and the startup of the monitoring system are presented.

### 3.1. Data Acquisition and Processing

The environmental variables to be monitored were identified in the early phase of data acquisition, through the review of the available scientific literature, and also according to the information supplied by local farmers to define the suitable climatic conditions for efficient tomato growth. As a result of this survey, we concluded that the variables shown in Table 2 are the most influential in the crop evolution [4,49,50,51]. The appropriate ranges were defined for each variable, as well as the possible negative effects that affect the crop when the actual values differ considerably from the suggested. Since the greenhouses under study are geographically located in an area with frequent and considerable winds (altitude about 2700 m), we included the wind speed and direction, because they are related to the air relative humidity, and hence to the greenhouse ventilation [52].

The sensors were selected based on the variables and measuring ranges presented in Table 2, and relevant technical criteria such as low power consumption, compatibility with processing cards, and linear response curve where achieved. Table 3 summarizes the main technical specifications of the sensors used for each variable.

**Figure 2:**
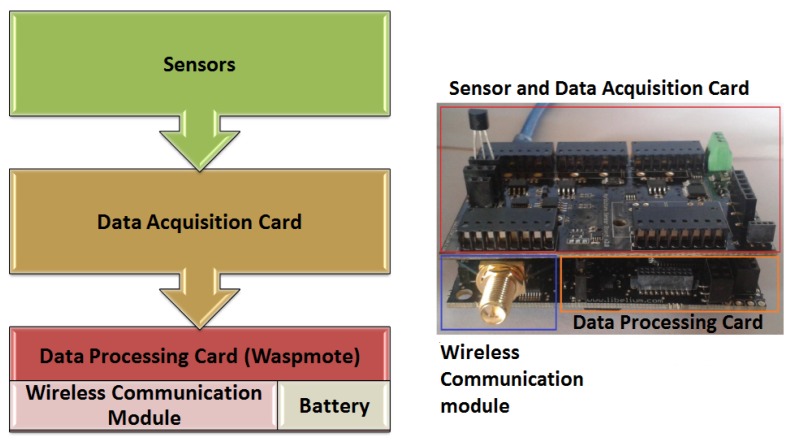
Structure and view of the sensor nodes.

The acquired measurements were processed through sensor nodes, whose structure is shown in Figure 2. They consisted of an agricultural or gas data acquisition card depending on the type of connected sensors, a data processing card (known as Waspmote), an omnidirectional antenna, a battery of 10 amperes per hour of load capacity, and a (ZigBee, DigiMesh, or WiFi) wireless communication module, according to the network type.

Data acquisition was performed using the agriculture PRO 2.0 card for measuring with sensors of air temperature, solar radiation, luminosity, air relative humidity, wind speed, wind direction, and UV radiation. Gases PRO 2.0 card was also used for the CO${}_{2}$ sensor. The energy consumption was approximately 106 micro amps. These cards were mounted on the processing card for recognition and configuration.

The Waspmote processing card was selected for the low power consumption, which is below seven micro amps, and also the modular architecture facilitated the integration of sensors and communication modules of different technologies and manufacturers. The data acquired by the sensors were stored in the internal memory of the Waspmote card in floating point format. The data acquired in the ZigBee and DigiMesh networks were multiplied by a constant to transform them into integer values of five positions, because this is a valid format for the communication modules. In the case of WiFi networks, this procedure was not required, since the transmitted frames accept both data types.

**Figure 3:**
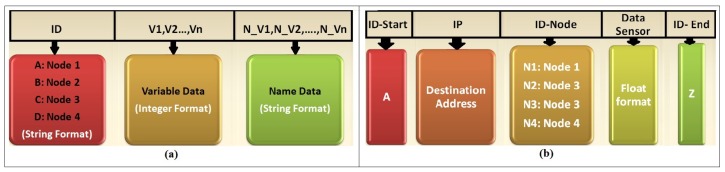
Structure of data transfer packages: (**a**) ZigBee and DigiMesh networks; and (**b**) WiFi Network.

The structure of the packages transferred from the Waspmote card to the communication modules of each network is shown in Figure 3. In the ZigBee and DigiMesh networks, the transfer was via UART serial controller, and it was necessary to create packages with the ID field using string format representing the node identifier. The next field is integer type and it corresponds to the measurements of each sensor. The last field is string type and it contains the name of each variable. For the WiFi network, the transfer was in frame format using the HTTP protocol. In each frame, it was specified the send start identifier, the destination IP address, the node identifier, the data of each sensor, and the send-end identifier. Both packaging techniques facilitated the reception and display of data processes.

### 3.2. Transmission and Reception of Packages for ZigBee and DigiMesh Networks

The XBEE communication modules were chosen to send and receive packages, since their cost and energy consumption are low. The ZigBee network was implemented with XBEE ZB S2 PRO modules, whose firmware allows creating networks with tree topology. In the case of the DigiMesh network, the XBEE ZB S1 PRO modules were used, because the firmware facilitates the configuration of networks with mesh topology. The XBEE modules were configured with the X-CTU software, which provides a friendly graphical user interface. For the communication between nodes to be successful, the XBEE modules were configured with the consideration that all modules operate in the same network group *PAN ID*, channel *CH*, and transmission *BD*. The parameters that were configured in each module are shown in Table 4. The source and destination addresses were assigned based on the serial number printed on the modules. Since the type of communication was broadcast, the destination address was the same on all nodes and corresponded to *DH = 13A200* and *DL = FFFF*. The transmission speeds *BD* were configured to different values for analysis of energy consumption and according to the operating rates of the Waspmote modules. Each network was configured in a different channel *CH* to avoid electromagnetic interference affecting the signals.

**Figure 4:**
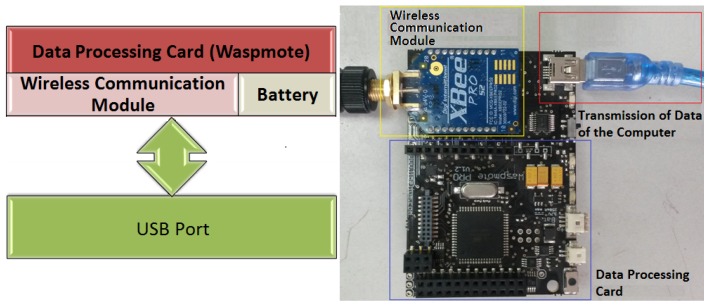
Structure of the coordinator nodes of the ZigBee and DigiMesh networks.

**Figure A1:**
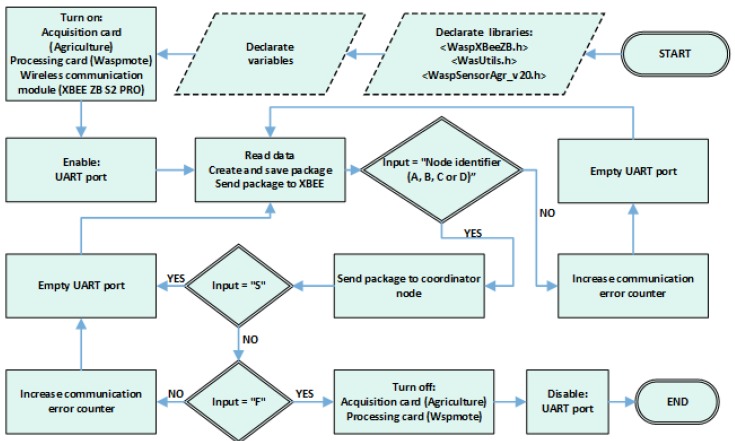
Flowchart of logic programming sensor nodes of the ZigBee network.

**Figure A2:**
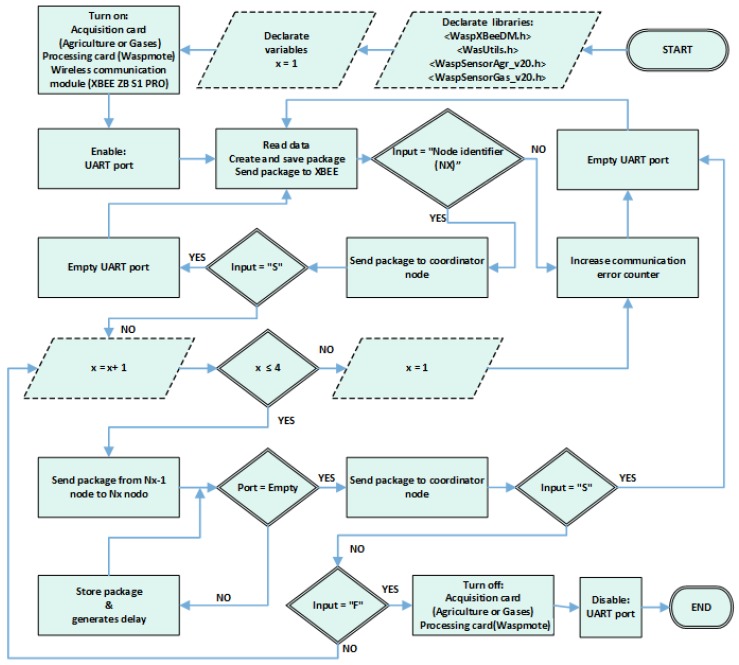
Flowchart of logic programming sensor nodes of the DigiMesh network.

**Figure A3:**
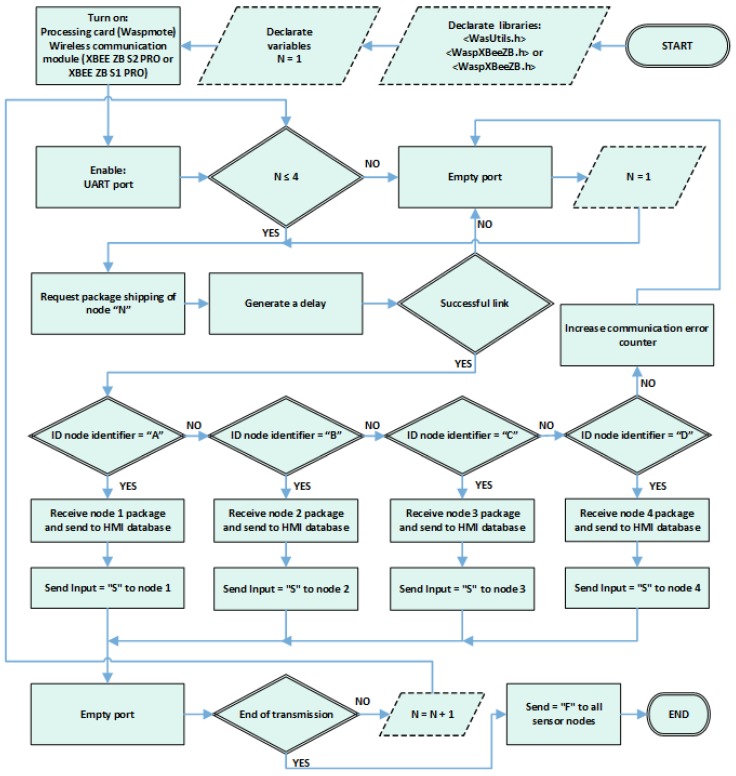
Flowchart of logic programming coordinator nodes of the of ZigBee and DigiMesh networks.

The programming code of the sensor nodes was developed in the ID PRO software of Libelium${}^{\mathrm{TM}}$. The logic that was used in the sensor nodes of both networks was similar, except for the routing process for sending data. Appendix A shows some technical details of the flowcharts of the programming of the sensor and coordinator nodes for the ZigBee and DigiMesh networks. The programming flowchart that was created for the sensor nodes of the ZigBee network is shown in Figure A1 (Appendix A). The flow diagram that was implemented for the DigiMesh network is shown in Figure A2 (Appendix A). The coordinating node structure shown in Figure 4 was implemented for the ZigBee and DigiMesh networks, and they differed only in the type of XBEE wireless communication module that they incorporated. This modules were also configured with the X-CTU software and they used the same parameters as in Table 3. The programming of the coordinating nodes of the ZigBee and DigiMesh networks were the same, and the flow diagram is shown in Figure A3 (Appendix A).

### 3.3. Transmission and Reception of Packages for WiFi Network

The transmission and reception of WiFi network packages was designed using a client–server architecture, where the sensor nodes are the clients and the coordinating node is the server. The packages of the sensor nodes of the WiFi network were transmitted to the coordinator through the RN-XV wireless communication module. The module was selected for its low power consumption, and it is also ideal for migrating from an 802.15.4 architecture network to TCP/IP. If a specific application is configured with XBee modules, and if it needs to be adapted to a WiFi network, then we simply need to configure the communication module and replace it on the Waspmote card, without changing the hardware architecture. The coordinator node corresponds to the Libelium Meshlium${}^{\mathrm{TM}}$ router, which is compatible with wireless technologies such as ZigBee and Bluetooth. It has several programming environments that are based on Linux, and it stores the data directly in a MySQL database. The relevant parameters configured in the sensor and the coordinator nodes are identifier network (SSID), protocol, password, IP address, communication port, and type of security. For the configuration of the RN-XV modules, we used Arduino Mega 2560 and Xbee Shield. In this process, the microprocessor of the Arduino card was disabled by placing a bridge between GND and the board reset. The Meshlium${}^{\mathrm{TM}}$ router was configured in the Manager System software. The access to the router is via the RJ 45 network port and is accessed with the IP address designated by default 10.10.10.1. The received data are automatically stored in a database with the start up time, and node identifier. This device also allows identifying all the clients (sensor nodes) that are connected, which facilitates the network management. Sending and receiving packages on the WiFi network is simple compared to the other two networks, since its configuration is similar to that of a WiFi network for domestic use. The potential offered by the router facilitates the data reception and storage.

### 3.4. HMI Design

Graphical interface was developed in LabVIEW${}^{\mathrm{TM}}$, which is a well known platform and development environment of virtual instruments (VI). The programming language was type G (Graphic/visual) for monitoring and control applications. LabVIEW${}^{\mathrm{TM}}$ enables a convenient connection to the Internet, which provides an ideal resource for real-time data acquisition and monitoring [53]. The developed graphical interface was similar for the three networks, with back-end differences in the communication link, its coordinator nodes, and in the software, as well as the data acquisition method.

**Figure 5:**
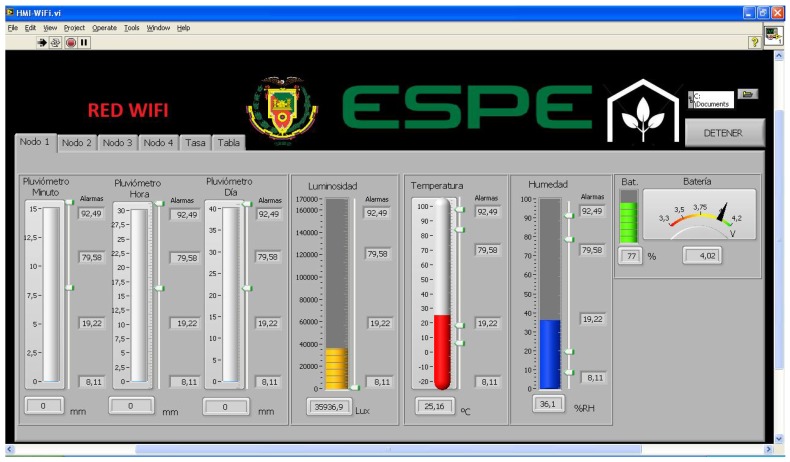
Monitoring of greenhouse variables, battery condition, alarms, data rate, and table for the WiFi network.

DigiMesh and ZigBee networks transfer data to LabVIEW${}^{\mathrm{TM}}$ via COM (USB); hence, they were stored in byte format in a software created table. The table was initialized for stored data, and the transmission speed was set at different values for the analysis of energy consumption. WiFi network was wirelessly linked to the computer, and the information was stored in the database that is automatically created in the Meshlium${}^{\mathrm{TM}}$ router. The access to data was done by creating a link between Meshlium${}^{\mathrm{TM}}$ and LabVIEW${}^{\mathrm{TM}}$. The MYSQL ODBC driver allowed the access to the database from any Windows application. The database was read and stored in LabVIEW${}^{\mathrm{TM}}$ through toolkits for creating, opening, and closing the communication channel, as well as for calling and storing data tables. Once data were available in LabVIEW${}^{\mathrm{TM}}$, the stages of design of the interface considered for the three networks corresponded to data separation, error detection, alarm generation, and variables monitoring. The data were separated by identifiers A, B, C, and D, for nodes 1, 2, 3, and 4, respectively, with LabVIEW${}^{\mathrm{TM}}$ case structures. The data were subsequently converted into floating numbers dividing them by a constant of 10,100 or 1000. Finally, data were entered and stored in a table with date, sensor node, and magnitude. The letters indicated that data were not received from the sensor node that produced the error. The design of alarms was done with normal ranges for tomato growing described in Table 2. The data variables displayed in the HMI were designed for each network. The program was able to indicate the HMI of the three networks according to user manipulation. Figure 5 shows some parts of the monitoring screen, such as greenhouse variables, battery condition, alarms, data rate, and table. The developed interface was user-friendly and allowed the user to know in real time and with accuracy the values of each variable, and to apply timely corrective actions in the greenhouse.

### 3.5. Mobile Application Development

The objective of the mobile application was that the environmental measures acquired by the three WSNs could be accessible from any geographical location by means of smartphones or tablets. The variables may be viewed in real time (trend), and through tables or graphs of a determined time interval (historical). In recent years, the most popular platforms in the mobile devices market are Android, Windows Phone and iOS, whose features were evaluated to select the most suitable for this application [54]. The comparative analysis of these platforms was oriented to aspects such as software architecture, accessibility to Web application programming environments, platform capabilities, and limitations. According to [55], Android is the platform with the best features to develop apps for mobile devices. On this basis, our Mobile Application was designed to be accessible from a device with Android Version 4 or higher operating system.

**Figure 6:**
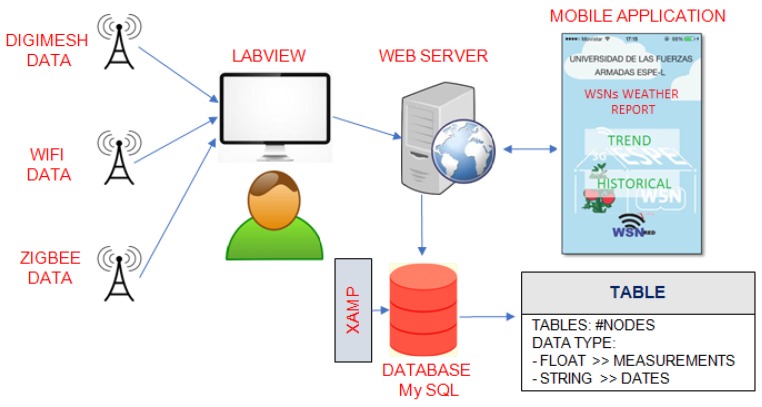
Mobile application development.

Figure 6 shows the main phases of the mobile application. The data acquired by the three networks were previously stored in LabVIEW${}^{\mathrm{TM}}$, and subsequently transferred to the Apache Web Server through the MySQL Connector/ODBC driver [56]. The data in the web server were distributed in tables using the multi-platform XAMPP software [57]. For each node of the three WSNs, we configured a table with floating data type for the environmental measurements and string for the dates. If a client (smartphone or tablet) requests the server, a PHP file is immediately executed, which contains the script code to manage the connection and to access to the MYSQL databases [58]. Every query type (trend or historical) was associated to a PHP script.

**Figure A4:**
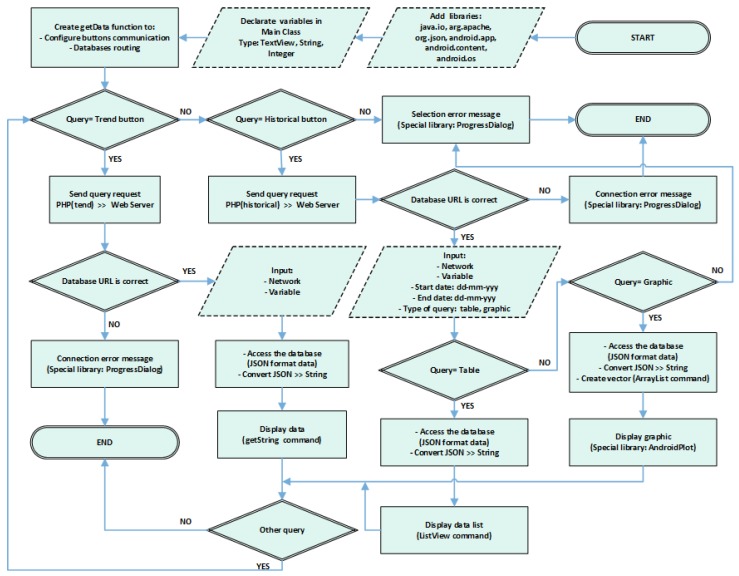
Flowchart of logic programming mobile application.

The web application was designed in the Eclipse Integrated Development Environment (IDE), currently considered the most popular framework among programmers. In addition, we used the software development kit (SDK) for the Android environment. The development of the application was divided into two parts. The first one was the design of the user interface through the configuration of graphical layouts in an XML script. The name, color, size, location and functionality of the interface buttons were assigned in this file. The second part was the programming of the buttons using JAVA to establish the communication between them, as well as the data visualization in real time or historical reports. Figure A4 (Appendix A) shows the flowchart of the programming logic developed for the mobile application.

### 3.6. Startup

**Figure 7:**
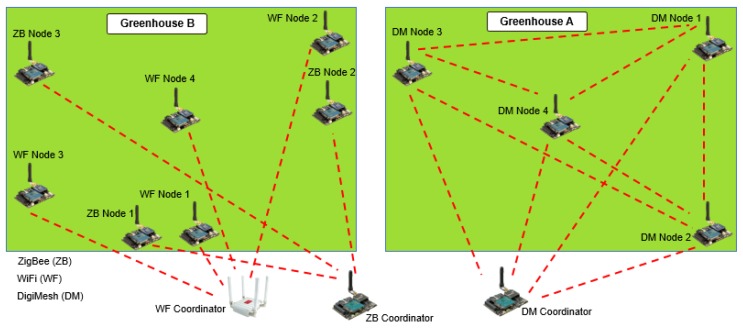
Map of WSNs distribution and node locations.

**Figure 8:**
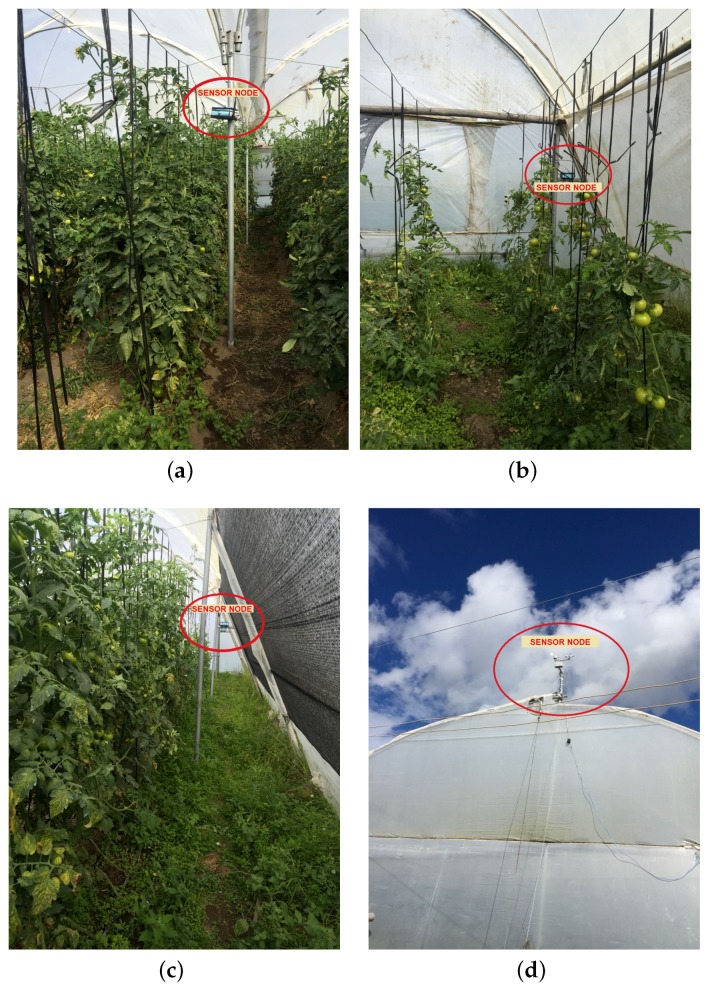
WSNs test scenario. (**a**) Node 4 of WiFi network; (**b**) Node 2 of WiFi network; (**c**) Node 1 of DigiMesh network; (**d**) Node 1 of ZigBee network.

The experiments took place in Salcedo town, located at coordinates −1.018373, −78.583888 in Ecuador. The agrarian area that corresponds to this study was 100 × 150 m and it is usually dedicated to the tomato cultivation. Our WSNs were implemented in two greenhouses, here denoted as A and B, with slightly different characteristics shown in Table 5. DigiMesh network was implemented in greenhouse A, and ZigBee and WiFi networks in greenhouse B. Sensor Nodes 1, 2, and 3 were installed at the ends of the greenhouses for variables air temperature, air relative humidity, luminosity, and radiation. The gas sensor in Node 4 was installed in the center to register the highest concentration of CO${}_{2}$ overnight, and the coordinator node was located at 30 m from the greenhouse. Figure 7 depicts the distribution networks and the location of the sensors and coordinator nodes, and Figure 8 shows the scenario where the networks were deployed and the location of some sensor nodes in the two greenhouses.

Once the networks were implemented, the startup of the LabVIEW${}^{\mathrm{TM}}$ graphical application was executed. Data were collected during three consecutive months, and the user was able to consult the real-time graphs of the variables of each node individually when needed. These databases were also used to analyze the power consumption of the three networks (in terms of different data transmission rates), the operation mode of wireless communication modules, and the distances among sensors and nodes of each network coordinators. Note that the designed system can be readily scaled to larger areas.

## 4. Results

According to preliminary experience during the configuration and implementation process of the three WSNs, we considered that some of relevant parameters for the performance analysis of each network are the complexity of configuration of a node to an eventual network expansion, and the data transmission speed. In addition, the dynamics of the energy consumption of each node and of the monitored variables were studied with detail, but their analysis is further discussed in the companion papers [33,34].

### 4.1. Node Configuration Complexity in the Case of Network Scalability

Based on the structure of each network, it was determined that the design of the DigiMesh network was the most complex, since the mesh topology was configured by redundant links between sensor nodes, and their programming included a more lines of code. Considering that this network does not include the option of link auto-configuration (ad-hoc), the addition of an element in the network would require extensive programming not only in the new node, but also in the existing ones. In the case of the ZigBee network that was configured with the star topology, redundancy does not exist, so fewer lines of code are used and their configuration complexity is reduced. However, if the link between the sensor node and its coordinator fails, the data would be permanently lost. The difficulty to add nodes would be intermediate, since the programming and configuration is executed in the new node and in the coordinator, but the rest of elements of the network are not modified.

The WiFi network was configured as Access Point (AP) topology, so that the clients (sensor nodes) were linked only to the Meshlium${}^{\mathrm{TM}}$ router, and they did not communicate with each other. The difficulty in programming the sensor nodes was lower compared to the other networks, since the data transfer uses the IP protocol, the frame of each transmission is created automatically, and it is not necessary to pack the data with the technique used in the ZigBee and DigiMesh networks. The configuration of the coordinating node was simple, since being a WiFi router, and the parameters were set as in the creation of any domestic use network. The data received in the router were automatically stored in a database, through the software that includes the device, a feature that facilitates data processing. In the other two networks, data acquisition is more complex because unpacking techniques were initially required, and then the database was created with additional software. The degree of complexity when adding a new element to the network would be relatively low, since the configuration of the existing nodes does not change.

### 4.2. Data Transmission Speed

The information transfer speed of each sensor node was an important parameter in the analysis of the performance of the three networks, since any communication system it is required to transmit as much information in a short time, without any possibility of data loss and with minimal error rate. The transmission rate was determined from the bit rate which is defined as the number of bits received by the coordinating node in a time interval. The binary speed is a parameter that varies according to the type of communication protocol, the number of nodes in the network, and the type of sensors installed in each of them. This parameter was obtained by programming LabVIEW${}^{\mathrm{TM}}$.

The number of bits received by the coordinators of the DigiMesh and ZigBee networks was updated in an average of three seconds. However, in the test phase, we identified that this time increased between 10 s and 20 s for the DigiMesh network when one or several sensor nodes were inactive, and therefore the bit rate was reduced. This effect was caused by the redundant links configured on this network. The update time between each packet received in the WiFi was around 8 s, because as explained in the previous sections, the coordinator node is a Meshlium${}^{\mathrm{TM}}$ router, and the preliminary data processing was carried out in the device software and later transferred to LabVIEW${}^{\mathrm{TM}}$. The upload time between the receipts of every data is larger in the WiFi network, because, as explained, the coordinating node of this network is a Meshlium${}^{\mathrm{TM}}$ device, the same that before establishing communication with LabVIEW${}^{\mathrm{TM}}$ performs the data preprocessing, hereby generating a higher update time between each reading.

The transmission rate $S_{T}$ of the three networks was calculated in LabVIEW${}^{\mathrm{TM}}$ through the quotient between the total bit rate $B_{T}$ and the update time of each measure $T_{M}$. This parameter was analyzed with all the active nodes and with some of them out of service. The results of $S_{T}$ for the three networks are shown in Table 6. These values revealed that the failure of one or more nodes of the ZigBee and WiFi networks does not influence the transmission speed, since its topology does not include the possibility of linking between sensor nodes. In the Digimesh network with mesh topology, the data transmission rate decreases as the number of inactive nodes increases, due to the redundant links that were configured in each network element.

From the tests performed at different heights of the sensor nodes, we conclude that this parameter affects the transmission speed. The results are observed in Table 7. For heights up to one meter, the bit rate of the WiFi and ZigBee networks decreased compared to the results shown in Table 6. This is because the height of the tomato crop in some cases exceeded one meter, losing line of sight with the coordination node, and in these circumstances some data were lost and the bit rate decreased. Redundant links from the DigiMesh network decreased the probability of data loss and the bit rate remained almost constant.

## 5. Discussion and Conclusions

This paper scrutinizes the design and implementation of three WSNs for the monitoring of environmental variables of interest in tomato greenhouses. Two networks used ZigBee technology, and they had different topologies (star and mesh), whereas, in the third, network WiFi technology with access point topology was used. The sensor nodes were classified according to the acquisition card used. Agricultural nodes were equipped with transducers of air temperature, air relative humidity, wind direction and speed, solar and UV radiation, and luminosity, while the gas nodes included a CO${}_{2}$ concentration sensor. The elements that integrated each node were configured in a low level programming language, except for the coordinator of the WiFi network that corresponded to the Meshlium${}^{\mathrm{TM}}$ router, whose network parameters were set in the device software. The coordinating nodes collected and sent measurements of the variables to a database configured on the system computer. The data were visualized with HMI designed in LabVIEW${}^{\mathrm{TM}}$, and with the mobile application created for smartphones with Android operating system. The networks were installed and put into operation in two greenhouses, and they were tested over three months. Their behavior was studied during this time interval by considering aspects such as scalability, changes of data transmission speed, error rate, and relation between the bit rate and the location node. In addition, this study has been the basis for two separate works, which describe in detail the dynamics of energy consumption measurements [33], and the dynamics of monitored environmental variables [34], acquired in each node of the three networks.

From the experience gained during the design, implementation, and startup of each network, we can conclude that the DigiMesh network is complex to redesign, because, to add a new sensor node, it is necessary to modify the programming of all the network elements. In the case of ZigBee network, the complexity is average, because it involves the configuration of the new nodes and of the coordinator. The difficulty of scalability of WiFi network is minimal, as the addition of new clients (sensor nodes) is performed by configuring the network identifier (SSID), and the parameters of the coordinator node do not need to be modified.

Regarding the bit rate, we obtained that it was greater for the WiFi network due to the type of IP communication protocol used by the coordinating node and the preliminary phase of data processing that is executed in the Meshlium${}^{\mathrm{TM}}$ device software, and these factors increase the time update between received packets. In the event that a node in the DigiMesh network is out of service, the bit rate will decrease considerably, because redundant links will be required and the data refresh time in the coordinator will be higher. This feature minimizes the possibility of data loss, but it also slows down the transmission rate.

Finally, we have shown that the location of the sensor nodes and the height of the crops strongly influence the data transmission rate, especially if the line of sight with the coordinator has been lost. The sensor nodes of the three networks were located at a lower height than the tomato crops, and we checked that the bit rate decreases considerably in networks with star topology. In the network with mesh topology, redundant links avoid data loss, so that the bit rate is not significantly affected. Our analysis of the different communication configurations allows determining the optimal parameters for the deployment of these types of networks within greenhouses. This research can be further extended with the development of a Machine learning system that allows to characterize and predict the behavior of the environmental variables registered by the WSNs. Specifically, in the future, we plan to apply our study to cooperate in research related to frost prediction in scenarios of the Andean region dedicated to large-scale cultivation, since this is a climatic factor that is unpredictable and it represents a major threat to crops. 

## Figures and Tables

**Table 1:** Summary of related work for WSNs in agriculture.

Geographical Location, Case Study, and Cultivation Area	Monitored Variables	Communication Technology	Network Topology	Store and Visualization Data	Objective
Iwata-Center Japan Tomato greenhouse (not specified area) [13]	Air temperature Luminosity Air relative humidity	Wireless ZigBee 2.4 GHz and 400 MHz	Star (3 sensor nodes)	Not specified	Comparative study of quality wireless communication in 400 MHZ and 2.4 GHz bands
Cotopaxi-Ecuador Rose greenhouse (50 m${}^{2}$) [27]	Air temperature Air relative humidity CO${}_{2}$ Soil moisture Luminosity	Wireless ZigBee 2.4 GHz	Star (3 sensor nodes)	LabVIEW${}^{\mathrm{TM}}$	Hardware and software development Data analysis of variables
Southern Italy Tomato greenhouse (100 m${}^{2}$) [41]	Air temperature Air relative humidity	Wireless ZigBee 2.4 GHz	Mesh (6 sensor nodes)	LabVIEW${}^{\mathrm{TM}}$	Hardware and software development Data analysis of variables
Chongqing-Center China Vegetables greenhouse (424.4 m${}^{2}$) [37]	Air temperature Luminosity Air relative humidity CO${}_{2}$	Wired Modbus	Bus (1 master—1 slave)	C&S System	Hardware and software development Data analysis of variables
Hubei-North China Vegetables greenhouse (100 m${}^{2}$) [42]	Air temperature Luminosity Air relative humidity	Wireless ZigBee 2.4 GHz	Star (20 sensor nodes)	Mobile app	Hardware and software development Data analysis of variables
Narpio-Western Finland Vegetables greenhouse (1440 m${}^{2}$) [43]	Air temperature Luminosity Air relative humidity CO${}_{2}$ Solar irradiance	Wireless ZigBee 2.4 GHz	Star (4 sensor nodes)	Not specified	Hardware and software development Data analysis of variables
Perlis-Nortwets Malasia Mango garden (520 m${}^{2}$) [44]	Air temperature Air relative humidity CO${}_{2}$	Wireless ZigBee 2.4 GHz	Star (3 sensor nodes)	LabVIEW${}^{\mathrm{TM}}$	Hardware and software development Data analysis of variables
Mauritius-Africa Potato field (5000 m${}^{2}$) [45]	Air temperature Air relative humidity Luminosity Soil pH Soil moisture	Wireless WiFi	Tree (6 sensor nodes)	Java	Hardware and software development Data analysis of variables
Yingde-North China Tea plantation (not specified area) [46]	Air temperature Soil water content Air relative humidity	Wired / Wireless Ethernet and GPRS	Mesh (20 sensor nodes)	Not specified	Hardware and software development Data analysis of variables
Yucatán-Southeast Mexico Habanero pepper garden (482.4 m${}^{2}$) [47]	Air temperature Air relative humidity Soil moisture	Wireless ZigBee 2.4 GHz	Star (6 sensor nodes)	Arduino	Fuzzy logic control of the irrigation system
Colima-Mexico Not specified (Not specified area ) [48]	Air temperature Air relative humidity Soil moisture	Wireless LORA-CBF	Star (5 sensor nodes)	Not specified	Evaluate a new WSN applied in agriculture

**Table 2:** Compilation of the required climatic conditions for growing tomatoes.

Variable	Normal Ranges	Level	Effect
Air temperature	Day (20–25 ${}^{\circ }$C) Night (15–18 ${}^{\circ }$C)	Higher	It affects fruiting (fall flowers, limitation on the mincemeat) (>30 ${}^{\circ }$C), and bad fertilization of fruit and therefore evil fruit filling
		Less	Short blade syndrome and fertilization problems (<10 ${}^{\circ }$C)
Air relative humidity	50 y 60 %	Higher	Fruit cracking, difficulty fertilization, reduces the absorption of nutrients, and poor fruit set
		Less	Securing the pollen to the stigma of the flower, water stress, stomatal closure, and reductions photosynthesis
Soil moisture	50%	High	Accelerated growth in plants, slows ripening of fruits, and increases the relative humidity
		Low	Fruit cracking, and water stress
Solar radiation	0.85 MJ/m${}^{2}$	Excess	Burnt fruit and plants
		Decrease	Lost productivity
Luminosity	8–16 h	Higher	Higher crop biomass, and increased density of plants
		Less	Fall Flower, insufficient pollination, and fruit size smaller
CO${}_{2}$	500–2000 ppm	High	Best plant development, and increased productivity
		Low	Photorespiration

**Table 3:** Technical features of the used sensors.

Variable	Sensor	Model	Range of measuring	Output signal	Power consumption
Carbon dioxide	Solid electrolyte	TGS4161	350–10,000 ppm	Linear	5 mA
Wind direction	Wind vane	WS-3000	16 positions	Discrete	<300 μA
Air relative humidity	Capacitor polymer	808H5V5	0–100% RH	Linear	0.38 mA
Luminosity	Photoresist	LDR	0–130,000 lux	Exponential	0 μA
Solar radiation	Apogee quantum	SQ-110	410–655 nm	Pulses	0 μA
UV radiation	Photodiode	SU-100	250–400 nm	Sinusoidal	0 μA
Air temperature	Thermistor	MCP9700A	−40 ${}^{\circ }$C to +125 ${}^{\circ }$C	Linear	6 μA
Wind speed	Anemometer	WS-3000	0–240 Km/h	Digital	<400 mA

**Table 4:** Configuration parameters for the communication modules.

Module	Node	MAC	Destination	PAN ID	Channel	Baud Rate
XBee ZB Pro S2	Node 1	0013A20040B5B798	DL: 13A200 DH: FFFF	4321	18	9600 19,200 57,600
	Node 2	0013A20040B5B7C2				
	Node 3	0013A20040B5B794				
	Coordinator	0013A20040B5B339				
DigiMesh Xbee Zb Pro S1	Node 1	0013A20040BDA364		1234	C	
	Node 2	0013A20040BDA365				
	Node 3	0013A20040BBB3D7				
	Node 4	0013A20040BDA364				
	Coordinator	0013A20040BBB3FA				

**Table 5:** Characteristics of tomato greenhouses.

Feature	Greenhouse A	Greenhouse B
Type	Sawtooth	Curve
Dimensions	Height: 5 m, length 80 m, width 50 m.	Height 5 m, length 70 m, width 50 m.
Phenological state [59]	Flowering	Harvest
Crop	Bulky	Less bulky

**Table 6:** Network transmission speed.

Network	Sensor Node	Transmission Rate (Bits)	$B_{T}$ (Bits)	Approximate $T_{M}$ (Seconds)	$S_{T}$ (bps)
DigiMesh	Node 1	318	974	3 (All active nodes) 10 (1 Faulty node) 20 (2 Faulty nodes)	324.667 (All active nodes) 97.4 (Faulty node) 48.7 (2 Faulty nodes)
	Node 2	278			
	Node 3	318			
	Node 4	60			
ZigBee	Node 1	334	970	3 (All cases)	323.333 (All cases)
	Node 2	318			
	Node 3	318			
WiFi	Node 1	318	696	8 (All cases)	87 (All cases)
	Node 2	318			
	Node 3	318			
	Node 4	60			

**Table 7:** Bit rate vs. height sensor nodes.

Height (m)	$B_{T}$ (Bits)		
	DigiMesh	ZigBee	WiFi
0	974	625	984
0.5	974	625	984
1	974	625	984
1.5	974	970	1044
1.8	974	970	1044

## Appendix A

In this Appendix, we show the flow diagrams applied to nodes for the ZigBee and DigiMesh networks (Figure A1, Figure A2, Figure A3), and the mobile application (Figure A4). The programming logic of the WiFi network nodes is not shown, because the sensor node was programmed very similarly to the ZigBee network, and the coordinating node (Meshlium${}^{\mathrm{TM}}$ router) was configured using the device own interface.

The data from the sensor nodes were sent to the coordinating node only if the node identifier entry corresponded to one of the letters defined in the package creation. In the case of no coincidences, a communication error counter was implemented for the quantification of the transmission error rate. The disadvantage of the topology of this network was that, if one of the nodes loses communication with the coordinator, then it would be permanently out of service.

The mesh topology of this network was implemented through redundant links, where each sensor node can communicate directly with the coordinator or through another sensor node. The programming that was developed in the sensor nodes of this network is more complex; however, the advantage was the low probability that some node was out of service, thus reducing the transmission error rate.

The programming of the sensor nodes of both networks included two entries of type string as indicators of important events in the network. The *S* and *F* indicators are characters sent from the coordinator to the sensor nodes. The *S* character represented the acknowledgment of receipt of the packet and the authorization of emptying the data bus. The *F* character symbolized the closing of the communication and the deactivation of the sensor nodes.

The sensor nodes and coordinator nodes were linked by the question–response sequence. The coordinator sends a data request to each sensor node, which in turn transfers the packet. If the data are successfully received in the set timeout, then the coordinator sends a successful link confirmation through the *S* character, and it sequentially erases the bus and makes a new data request. Otherwise, the rate error counter is incremented. Packet transmission ends when the coordinator sends the *F* character to each sensor node.

The programming of the mobile application was conditioned by the appropriate configuration of the PHP (request for connection to the Web server), and using XML (design and functionality of the user interface buttons) files, as well as the proper databases routing of every network hosted on the server. The programming sequence was executed according to the type of request, which may be either trend (data in a single time slot) or historical (data from a time slot); in both cases, it was necessary to convert the JSON data (typical database format) to string to later be represented as a single character (real time data), table, or graphic, as required in each case.

## References

[1] (2015). INEC (Instituto Nacional de Estadística y Censos). http://www.ecuadorencifras.gob.ec//documentos/web-inec/Estadisticas_agropecuarias/espac/espac_2014-2015/2015/Presentacion%20de%20resultados%20ESPAC_2015.pdf.

[2] Lee H., Kim M.S., Jeong D., Delwiche S.R., Chao K., Cho B.K. (2014). Detection of cracks on tomatoes using a hyperspectral near-infrared reflectance imaging system. Sensors.

[3] Shrestha S., Deleuran L.C., Olesen M.H., Gislum R. (2015). Use of multispectral imaging in varietal identification of tomato. Sensors.

[4] Atherton J., Rudich J. (2012). The Tomato Crop: A Scientific Basis for Improvement.

[5] Suárez Barón J.C., Suarez Baron M.J. Monitoreo de variables ambientales en invernaderos usando tecnología ZIGBEE. Proceedings of the XLIII Jornadas Argentinas de Informática e Investigación Operativa (43JAIIO)-VI Congreso Argentino de AgroInformática (CAI).

[6] Hemraj S. Power estimation and automation of green house using wireless sensor network. Proceedings of the 5th International Conference on Confluence the Next Generation Information Technology Summit (Confluence).

[7] Roldán J.J., Joossen G., Sanz D., del Cerro J., Barrientos A. (2015). Mini-UAV based sensory system for measuring environmental variables in greenhouses. Sensors.

[8] Srbinovska M., Gavrovski C., Dimcev V., Krkoleva A., Borozan V. (2015). Environmental parameters monitoring in precision agriculture using wireless sensor networks. J. Clean. Prod..

[9] Hwang J., Yoe H. (2011). Study on the context-aware middleware for ubiquitous greenhouses using wireless sensor networks. Sensors.

[10] Duarte-Galvan C., Romero-Troncoso R.d.J., Torres-Pacheco I., Guevara-Gonzalez R.G., Fernandez- Jaramillo A.A., Contreras-Medina L.M., Carrillo-Serrano R.V., Millan-Almaraz J.R. (2014). FPGA-Based Smart Sensor for Drought Stress Detection in Tomato Plants Using Novel Physiological Variables and Discrete Wavelet Transform. Sensors.

[11] Kalaivani T., Allirani A., Priya P. A survey on ZigBee based wireless sensor networks in agriculture. Proceedings of the 3rd International Conference on Trendz in Information Sciences and Computing (TISC).

[12] Chang C.L., Huang Y.M., Hong G.F. (2015). Using a Novel Wireless-Networked Decentralized Control Scheme under Unpredictable Environmental Conditions. Sensors.

[13] Ibayashi H., Kaneda Y., Imahara J., Oishi N., Kuroda M., Mineno H. (2016). A Reliable Wireless Control System for Tomato Hydroponics. Sensors.

[14] Li D., Xu L., Tan C., Goodman E.D., Fu D., Xin L. (2015). Digitization and visualization of greenhouse tomato plants in indoor environments. Sensors.

[15] Roldán J.J., Garcia-Aunon P., Garzón M., de León J., del Cerro J., Barrientos A. (2016). Heterogeneous multi-robot system for mapping environmental variables of greenhouses. Sensors.

[16] Xiaoyan Z., Xiangyang Z., Chen D., Zhaohui C., Shangming S., Zhaohui Z. The design and implementation of the greenhouse monitoring system based on GSM and RF technologies. Proceedings of the International Conference on Computational Problem-solving (ICCP).

[17] Asolkar P.S., Bhadade U.S. An Effective Method of Controlling the Greenhouse and Crop Monitoring Using GSM. Proceedings of the International Conference on Computing Communication Control and Automation (ICCUBEA).

[18] Huang H., Bian H., Zhu S., Jin J. A Greenhouse Remote Monitoring System Based on GSM. Proceedings of the International Conference on Information Management, Innovation Management and Industrial Engineering (ICIII).

[19] Li X.H., Cheng X., Yan K., Gong P. (2010). A Monitoring System for Vegetable Greenhouses based on a Wireless Sensor Network. Sensors.

[20] Shi Y.B., Xiu D.B., Shi Y.P., Wang X., Wang M.M., Wang R.X. (2013). Design of Wireless Sensor System for Agricultural Micro Environment Based on WiFi. Appl. Mech. Mater..

[21] Mendez G.R., Yunus M.A.M., Mukhopadhyay S.C. A WiFi based smart wireless sensor network for monitoring an agricultural environment. Proceedings of the of International Conference on Instrumentation and Measurement Technology (I2MTC).

[22] Wang Y., Di W. Application of Wi-Fi Wireless Communication Technology In The Remote Monitoring of the Greenhouse. Proceedings of the 1st International Conference on Information Sciences, Machinery, Materials and Energy (ICISMME).

[23] Deng X., Zheng L., Li M. Development of a field wireless sensors network based on ZigBee technology. Proceedings of the World Automation Congress (WAC).

[24] Wang W., He G., Wan J. Research on ZigBee wireless communication technology. Proceedings of the International Conference on Electrical and Control Engineering (ICECE).

[25] Baviskar J., Mulla A., Baviskar A., Ashtekar S., Chintawar A. Real Time Monitoring and Control System for Green House Based on 802.15.4 Wireless Sensor Network. Proceedings of the 4th International Conference on Communication Systems and Network Technologies (CSNT).

[26] Farahani S. (2008). ZigBee Wireless Networks and Transceivers.

[27] Erazo M., Rivas D., Pérez M., Galarza O., Bautista V., Huerta M., Rojo-Álvarez J.L. Design and implementation of a wireless sensor network for rose greenhouses monitoring. Proceedings of the 6th International Conference on Automation, Robotics and Applications (ICARA).

[28] Pawlowski A., Guzman J.L., Rodríguez F., Berenguel M., Sánchez J., Dormido S. (2009). Simulation of Greenhouse Climate Monitoring and Control with Wireless Sensor Network and Event-Based Control. Sensors.

[29] Li L., Xiaoguang H., Ke C., Ketai H. The applications of WiFi-based Wireless Sensor Network in Internet of Things and Smart Grid. Proceedings of the 6th IEEE Conference on Industrial Electronics and Applications (ICIEA).

[30] Ruiz-Garcia L., Lunadei L., Barreiro P., Robla I. (2009). A Review of Wireless Sensor Technologies and Applications in Agriculture and Food Industry: State of the Art and Current Trends. Sensors.

[31] Jimenez A., Jimenez S., Lozada P., Jimenez C. Wireless Sensors Network in the Efficient Management of Greenhouse Crops. Proceedings of the 9th International Conference on Information Technology: New Generations (ITNG).

[32] Montoya F.G., Gomez J., Cama A., Zapata-Sierra A., Martinez F., De La Cruz J.L., Manzano-Agugliaro F. (2013). A monitoring system for intensive agriculture based on mesh networks and the Android system. Comput. Electron. Agric..

[33] Erazo-Rodas M., Sandoval-Moreno M., Muñoz-Romero S., Rivas D., Huerta M., Rojo-Álvarez J.L. (2018). Multiparametric Monitoring in Equatorian Tomato Greenhouse (II): Energy Consumption Dynamics. Sensors.

[34] Erazo-Rodas M., Sandoval-Moreno M., Muñoz-Romero S., Rivas D., Huerta M., Rojo-Álvarez J.L. (2018). Multiparametric Monitoring in Equatorian Tomato Greenhouse (III): Environment Measurement Dynamics. Sensors.

[35] Zhuang W., Zhi J., Hong L.G. Temperature and Humidity Measure-Control System Based on CAN and Digital Sensors. Proceedings of the International Forum on Information Technology and Applications (IFITA).

[36] Li X., Liu Q., Yang R., Zhang H., Zhang J., Cai E. (2015). The Design and Implementation of the Leaf Area Index Sensor. Sensors.

[37] Yang M., Li X., Yang R. Greenhouse Environment Control and Monitoring System in the Hilly Area of Chongqing. Proceedings of the International Conference on Intelligent Computation Technology and Automation (ICICTA).

[38] Yulong J., Jiaqiang Y. Design an Intelligent Environment Control System for GreenHouse Based on RS485 Bus. Proceedings of the 2nd International Conference on Digital Manufacturing and Automation (ICDMA).

[39] Hwang J., Shin C., Yoe H. (2010). A wireless sensor network-based ubiquitous paprika growth management system. Sensors.

[40] Ramírez M., Guadalupe L., Fuentes-Marilles O., García-Jiménez F. (2012). Heladas.

[41] Mancuso M., Bustaffa F. A wireless sensors network for monitoring environmental variables in a tomato greenhouse. Proceedings of the International Workshop on Factory Communication Systems.

[42] Huang Y. Design and Realization of Wireless Sensor Network for Vegetable Greenhouse Information Acquisition. Proceedings of the 6th International Conference on Wireless Communications Networking and Mobile Computing (WiCOM).

[43] Ahonen T., Virrankoski R., Elmusrati M. Greenhouse Monitoring with Wireless Sensor Network. Proceedings of the International Conference on Mechtronic and Embedded Systems and Applications (MESA).

[44] Saad S., Munirah Kamarudin L., Kamarudin K., Nooriman W., Mamduh S., Zakaria A., Md Shakaff A., Jaafar M. A real-time greenhouse monitoring system for mango with Wireless Sensor Network (WSN). Proceedings of the 2nd International Conference on Electronic Design (ICED).

[45] Khedo K.K., Hosseny M.R., Toonah M.Z. PotatoSense: A wireless sensor network system for precision agriculture. Proceedings of the IST-Africa Conference.

[46] Sun D., Jiang S., Wang W., Tang J. WSN Design and Implementation in a Tea Plantation for Drought Monitoring. Proceedings of the International Conference on Cyber-Enabled Distributed Computing and Knowledge Discovery (CyberC).

[47] Ceballos M., Gorricho J.L., Palma O., Huerta M., Rivas D., Erazo M. (2015). Fuzzy System of Irrigation to Applied to the Growth of Habanero Pepper (Capsicum chinese Jacq.) under Protected Conditions in Yucatan—México. Int. J. Distrib. Sens. Netw..

[48] Aquino-Santos R., González-Potes A., Edwards-Block A., Virgen-Ortiz R.A. (2011). Developing a new wireless sensor network platform and its application in precision agriculture. Sensors.

[49] Salazar R., Rojano A., Lopez I., Schmidt U. A Model for the Combine Description of the Temperature and Relative Humidity Regime in the Greenhouse. Proceedings of the 9th Mexican International Conference on Artificial Intelligence (MICAI).

[50] Nuez F. (1995). El Cultivo del Tomate.

[51] Rodriguez R., Rodriguez T. (2001). Cultivo Moderno del Tomate.

[52] Boulard T., Fatnassi H., Roy J., Lagier J., Fargues J., Smits N., Rougier M., Jeannequin B. (2004). Effect of greenhouse ventilation on humidity of inside air and in leaf boundary-layer. Agric. For. Meteorol..

[53] Alexander P.J., Radhakrishnan N. Remote lab implementation on an embedded web server. Proceedings of the International Conference on Circuit Power and Computing Technologies (ICCPCT).

[54] Grønli T.M., Hansen J., Ghinea G., Younas M. Mobile application platform heterogeneity: Android vs. Windows Phone vs. iOS vs. Firefox OS. In Proceedings of the 28th International Conference on Advanced Information Networking and Applications (AINA).

[55] Tiernan P. (2010). Enhancing the learning experience of undergraduate technology students with LabVIEW™ software. Comput. Educ..

[56] Kofler M. (2006). The Definitive Guide to MySQL 5.

[57] Martín A.R., Martín M.J.R. (2014). Aplicaciones Web.

[58] Olsson M. (2016). Using PHP. PHP 7 Quick Scripting Reference.

[59] CENTA (Centro Nacional de Tecnología Agropecuaria y Forestal). http://www.centa.gob.sv/docs/guias/hortalizas/Guia%20Tomate.pdf.

